# From Metagenomics to Discovery of New Viral Species: Galium Leaf Distortion Virus, a Monopartite Begomovirus Endemic in Mexico

**DOI:** 10.3389/fmicb.2022.843035

**Published:** 2022-04-25

**Authors:** Enrique A. Guevara-Rivera, Edgar A. Rodríguez-Negrete, Elva T. Aréchiga-Carvajal, Norma E. Leyva-López, Jesús Méndez-Lozano

**Affiliations:** ^1^Instituto Politécnico Nacional, CIIDIR-Unidad Sinaloa, Departamento de Biotecnología Agrícola, Guasave, Mexico; ^2^Universidad Autónoma de Nuevo León, Facultad de Ciencias Biológicas, Departamento de Microbiología e Inmunología-Unidad de Manipulación Genética, San Nicolás de los Garza, Mexico

**Keywords:** begomoviruses, non-cultivated plants, high-throughput sequencing (HTS), plant virome, new world (NW) monopartite begomovirus, geometagenomics

## Abstract

Begomoviruses (Family *Geminiviridae*) are a major group of emerging plant viruses worldwide. The knowledge of begomoviruses is mostly restricted to crop plant systems. Nevertheless, it has been described that non-cultivated plants are important reservoirs and vessels of viral evolution that leads to the emergence of new diseases. High-throughput sequencing (HTS) has provided a powerful tool for speeding up the understanding of molecular ecology and epidemiology of plant virome and for discovery of new viral species. In this study, by performing earlier metagenomics library data mining, followed by geminivirus-related signature single plant searching and RCA-based full-length viral genome cloning, and based on phylogenetic analysis, genomes of two isolates of a novel monopartite begomovirus species tentatively named *Galium leaf distortion virus* (GLDV), which infects non-cultivated endemic plant *Galium mexicanum*, were identified in Colima, Mexico. Analysis of the genetic structure of both isolates (GLDV-1 and GLDV-2) revealed that the GLDV genome displays a DNA-A-like structure shared with the new world (NW) bipartite begomoviruses. Nonetheless, phylogenetic analysis using representative members of the main begomovirus American clades for tree construction grouped both GLDV isolates in a clade of the monopartite NW begomovirus, *Tomato leaf deformation virus* (ToLDeV). A comparative analysis of viral replication regulatory elements showed that the GLDV-1 isolate possesses an array and sequence conservation of iterons typical of NW begomovirus infecting the *Solanaceae* and *Fabaceae* families. Interestingly, GLDV-2 showed iteron sequences described only in monopartite begomovirus from OW belonging to a sweepovirus clade that infects plants of the *Convolvulaceae* family. In addition, the rep iteron related-domain (IRD) of both isolates display FRVQ or FRIS amino acid sequences corresponding to NW and sweepobegomovirus clades for GMV-1 and GMV-2, respectively. Finally, the lack of the GLDV DNA-B segment (tested by molecular detection and biological assays using GLDV-1/2 infectious clones) confirmed the monopartite nature of GLDV. This is the first time that a monopartite begomovirus is described in Mexican ecosystems, and “*in silico*” geometagenomics analysis indicates that it is restricted to a specific region. These data revealed additional complexity in monopartite begomovirus genetics and geographic distribution and highlighted the importance of metagenomic approaches in understanding global virome ecology and evolution.

## Introduction

Viruses belonging to the *Geminiviridae* family with over 520 species constitute the major group of emerging plant viruses affecting a multitude of important vegetable and fiber crops and causing significant economic losses in many countries located in tropical and subtropical regions of the world (Varma and Malathi, 2003). Geminiviruses possess small single-stranded DNA genomes packed in twin quasi-icosahedral virions. The *Geminiviridae* family is classified into 14 genera recognized by the International Committee of Taxonomy of Viruses (ICTV), *Becurtovirus*, *Begomovirus*, *Capulavirus*, *Curtovirus*, *Eragrovirus*, *Grablovirus*, *Mastrevirus*, *Topocuvirus*, *Turncurtovirus*, *Citlodavirus*, *Maldovirus*, *Mulcrilevirus*, *Opunvirus*, and *Topilevirus*, according to its genome organization, host range, insect vector, and phylogeny (Fiallo-Olivé et al., 2021). Begomoviruses, vectored by the polyphagous *Bemisia tabaci* complex, are the largest (> 400 recognized members) and ecologically more successful genus in the entire virosphere infecting a wide range of dicotyledonous plants (Navas-Castillo et al., 2011). Begomovirus genomes are either monopartite or bipartite depending on the presence of one or two components referred to as DNA-A and DNA-B ranging between 2.6 and 2.8 kb in size. DNA-A encodes the AV1/CP coat protein in a virion sense orientation, and four genes (AC1/Rep, AC2/TrAP, AC3/REn, and AC4) in a complementary sense, which are involved in viral DNA replication, plant cell cycle regulation, and host defense suppression. On the other hand, DNA-B displays in a virion sense orientation the BV1/NSP gene and in a complementary sense orientation the BC1/MP gene involved in cell-to-cell and systemic viral movements, respectively. The genome organization of monopartite begomovirus is similar to bipartite DNA-A (Fondong, 2013; Hanley-Bowdoin et al., 2013). The genomic components of begomoviruses share low homology with the exception of a ∼200-nt known common region (CR) located within the intergenic region that includes a replication origin where the Rep protein, by binding to reiterated motifs (iterons), initiates viral DNA replication and introduces a nick in the conserved nanonucleotide TAATATTAC (Argüello-Astorga et al., 1994). Begomoviruses are phylogenetic and geographically divided (based on DNA-A sequences) into four lineages: old world (OW) begomoviruses (Africa, Eurasia, Oceania, and Indian subcontinent), new world (NW) begomoviruses (Latin and Mesoamerica), sweepoviruses (America, Asia, and Europe), and legumoviruses (India and Southeast Asia) (Rybicki, 1994; Briddon et al., 2010). OW begomoviruses are predominantly monopartite and are commonly associated with satellite DNA molecules (alpha, beta, and delta), which are involved in pathogenicity (Lozano et al., 2016; Briddon et al., 2018; Yang et al., 2019), whereas most NW begomoviruses (∼140 species) possess bipartite genomes, with few exceptions (Melgarejo et al., 2013; Sánchez-Campos et al., 2013; Vu et al., 2015; Macedo et al., 2018; Romay et al., 2019; Fiallo-Olivé and Navas-Castillo, 2020). Sweepoviruses are exclusively monopartite (Abdel-Salam et al., 2016), and legumoviruses possess bipartite genomes (Alabi et al., 2010). Recent studies have shown that viral genetic structure and geographic distribution, which rule OW and NW distinction, are more complex than were thought. After the OW monopartite *Tomato yellow leaf curl virus* (TYLCV) was introduced into the Americas in the early 1990s, indigenous NW tomato-infecting monopartite begomoviruses including *Tomato leaf deformation virus* (ToLDeV) in Ecuador and Peru (Melgarejo et al., 2013; Sánchez-Campos et al., 2013), *Tomato mottle leaf curl virus* (ToMoLCV), *Tomato leaf curl purple vein virus* in Brazil (Vu et al., 2015; Macedo et al., 2018), and *Tomato twisted leaf virus* (ToTLV) in Venezuela (Romay et al., 2019) have been described. Additionally, an NW monopartite begomovirus named *Corchorus yellow vein Cuba virus* (CoYVCUV) infecting the non-cultivated plant *Corchorus siliquosus* (family *Malvaceae*) in Cuba (Fiallo-Olivé and Navas-Castillo, 2020) was recently described. Begomoviruses show a high potential of genetic variation by accumulative mutations and recombination resulting in viral adaptation to new hosts and the emergence of new diseases (Elena et al., 2014). To date, studies on evolutive factors involved in begomovirus emergence have been restricted to a few pathosystems and geographic regions associated with cultivated plants. However, recently, it has been described that non-cultivated plants growing in an interface between crops and natural ecosystems could act as reservoirs and evolution vessels of both crop-adapted and non-cultivated-adapted begomoviruses. Therefore, the potential impact of the global viral genomic population (virome) in the emergence or re-emergence of plant diseases has been underestimated (García-Arenal and Zerbini, 2019). High-throughput sequencing (HTS) has provided a powerful tool for detecting known viruses and identifying novel plant viruses using metagenomics (viral genomic material contained in an environmental sample) and ecogenomics (viral genomic material contained in an individual sample) approaches (Roossinck, 2011; Barba et al., 2014; Massart et al., 2014; Stobbe and Roossinck, 2014; Wu et al., 2015; Rodríguez-Negrete et al., 2019). Mexico is considered one of the most megadiverse countries in the globe (Llorente-Bousquets and Ocegueda, 2008) with a rich variety of several endemic non-cultivated plant species with the potential to harbor an unexplored virome. In this study, by using earlier metagenomic library data mining followed by geminivirus-related signature single plant searching and RCA-based full-length viral genome cloning, a novel NW monopartite begomovirus species from a non-cultivated plant endemic in Mexico is first reported.

## Materials and Methods

### Metagenomic Data, Plant Samples, and Begomovirus Signature Detection

The metagenomics data used in this study have been previously described (Rodríguez-Negrete et al., 2019), and contigs containing geminivirus-related signatures are available at https://www.dropbox.com/sh/ha6pkzls9217dhf/AAADNUa0TfYj3EZ8bb315cSga?dl=0. Node 17 from the Colima-Nayarit library contains a contig of 465 bp and a geminivirus-related signature showing an 80% similarity with the begomovirus *Sweet potato leaf curl virus* (SPLCV) from China (accession number: MH602265) was selected for further analysis. The SPLCV-like signature was used as a template to design a primer set (SP-Like-F: TCGAACTGCACAAGCACATG/SP-Like-R: AGCTCAGAGTTGGTGACATCC, expected amplicon: 232 bp) using the PrimerSelect (DNASTAR Inc., Madison, WI, United States) software. For identification of plants harboring the SPLCV-like signature, a total of 49 plants belonging to nine families (*Amaranthaceae*, *Asteraceae*, *Convolvulaceae*, *Cucurbitaceae*, *Malvaceae*, *Nyctaginaceae*, *Rubiaceae*, *Solanaceae*, and *Verbenaceae*) from the Colima state sample collection (Rodríguez-Negrete et al., 2019) were individually analyzed. Total DNA from frozen plant tissues was extracted using the CTAB-based method (Doyle and Doyle, 1987), and 100 ng of DNA was used as a template for PCR detection with the SP-Like-F/SP-Like-R primer set.

### Cloning of Viral DNA and Sequence Analysis

To obtain putative full-length begomovirus genomes, total DNA from a selected SPLCV-like signature-positive sample was amplified by rolling circle amplification (RCA) with φ-29 DNA polymerase (TempliPhi; GE Healthcare) as described previously (Inoue-Nagata et al., 2004). The resulting concatemers were digested independently with two different restriction enzymes (*Bam*HI, and *Sac*I), yielding one fragment of 2.7 kb and two fragments of 1.8 and 0.9 kb. The three fragments were cloned into a pGreen 0029 vector (Hellens et al., 2000) and transformed in *E. coli* DH5-α, and two independent clones of each fragment were sequenced in both directions with the Sanger method using M13 forward and reverse primers, and then with the primer walking strategy by specific primer design when necessary. Sequence assemblies were obtained using the SeqMan (DNASTAR Inc.) program. The comparisons of complete circular genomes and their individual open reading frames (ORFs) were performed with MEGA 7.0 software (Kumar et al., 2016) and compared with the highest match viral genome homologous available in the NCBI databank. To validate original metagenomics data, full-length begomovirus genomes obtained by RCA-based cloning were aligned with HTS read libraries (Rodríguez-Negrete et al., 2019) with Bowtie2 (Langmead and Salzberg, 2012) using the Galaxy server^1^ and visualized with Integrated Genome Browser 9.0.2 (Freese et al., 2016)^2^.

### Phylogenetic and Recombination Analysis

Phylogenetic trees were constructed using the maximum likelihood method. For nucleotide sequence-based trees, selected DNA-A sequences were aligned in MEGA 7 using the MUSCLE method (Edgar, 2004) and the GTR (R + I) model with a bootstrap test of 1,000 replicates. For amino acid sequence-based trees, MEGA 7 alignments were performed, and the JTT + G model with 1,000 repetitions of bootstrap was used. For amino acid sequence comparison, alignments were performed with Clustal Omega^3^ and protein motifs analyzed manually. Recombination analysis was performed with the RDP4 program (Martin et al., 2015) using default settings and all recombination detection methods, RDP, GENCONV, BookScan, MaxChi, SiScan, Chimera, and 3Seq, followed by alignment of selected sequences with the MUSCLE program (Edgar, 2004).

### Begomovirus Regulatory Element Analysis

Regulatory begomoviral elements involved in replication and transcription have been described previously (Argüello-Astorga et al., 1994; Argüello-Astorga and Ruiz-Medrano, 2001; Mauricio-Castillo et al., 2014; Cantú-Iris et al., 2019). Intergenic regions of selected begomoviruses were visualized with the SnapGene (GLS Biotech, LLC) software, and regulatory elements were identified by visual inspection.

### Galium Leaf Distortion Virus Infectious Clone Construction

Infectious begomovirus partial dimeric clones of *Galium leaf distortion virus* (GLDV) isolates (GLDV-1 and GLDV-2) were constructed as previously described (Bang et al., 2014). A fragment of 1.5 kb was released from monomeric constructs pG-GLDV-1 and pG-GLDV-2 by *Bam*HI and *Sac*I double digestion and cloned in a pGreen 0029 binary vector linearized with same enzymes to yield pG-GLDV-1 0.6mer and pG-GLDV-2 0.6mer constructs, respectively. GLDV-1 0.6mer and pG-GLDV-2 0.6mer intermediaries were linearized with *Bam*HI and dephosphorylated with shrimp alkaline phosphatase (rSAP) (New England BioLabs) following the manufacturer’s instructions. Then, the fragments were ligated with a monomeric copy of a full-length viral genome released with *Bam*HI from the corresponding monomeric construct to yield GLDV-1 1.6mer and pG-GLDV-2 1.6mer constructs. In each cloning step, positive clones were verified by enzymatic digestion. Positive partial dimeric clones were transformed into *Agrobacterium tumefaciens* GV3101 strain by electroporation (25 μF, 400 Ω, and 2,500 V) in a Gene PulserXCell (Bio-Rad) apparatus.

### Plant Inoculation and Begomovirus Detection

For inoculation of GLDV-derived infectious clones, *Agrobacterium tumefaciens* strains harboring partial dimeric constructs were grown and inoculated as previously described (Cañizares et al., 2015). Plant species used for agroinoculation assays were *Nicotiana benthamiana*, tomato (*Solanum lycopersicum*), Hybrid Maya. The plants were agroinoculated in the four-leaf stage by stem puncture and leaf infiltration, maintained for 4 days in a bioclimatic chamber, BINDER, at 24*^o^*C under long-day conditions (16/8 light/darkness), and, after that, transferred to a greenhouse. Symptoms were documented weekly, and samples of non-inoculated new emerging apical leaves were collected 21 days post-inoculation (dpi). Begomovirus molecular detection was performed by PCR using *Galium leaf distortion virus* (GLDV)-specific primers recognizing both viral isolates, designed to amplify a fragment of viral intergenic regions (IR GLDV-F: CCGCAGGGGGACAGATAAG/IR GLDV-R: AAGTGAATGAATTATTGCGGGC, expected amplicon: 201 bp). Infection assays were performed in three independent biological replicates of 3-4 plants each.

## Results

### The Non-cultivated Plant *Galium mexicanum* Is a Reservoir of a Novel Begomovirus Species

**FIGURE 1:**
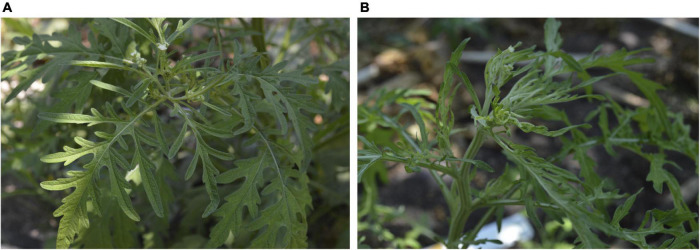
*Galium mexicanum* plants sampled in Tecomán, Colima, México. **(A)** Plant with a symptomless appearance. **(B)** Plants exhibiting apical leaf distortion symptoms.

**FIGURE 2:**
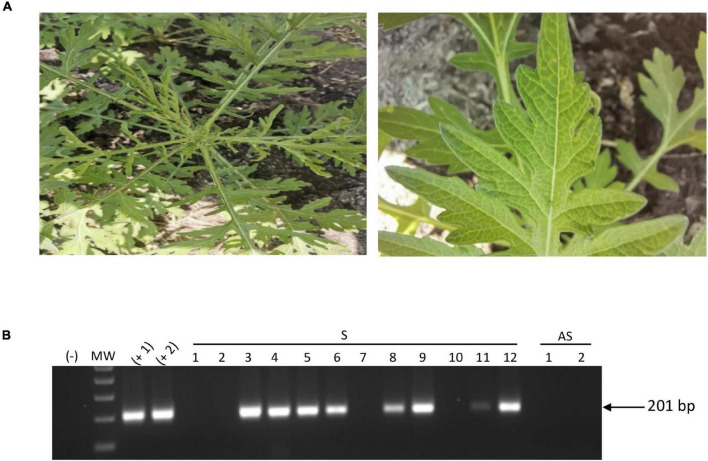
Incidence of *Galium leaf distortion virus* (GLDV) in Tecomán, Colima region. **(A)** Samples of *Galium mexicanum* showing leaf distortion and foliar chlorosis symptoms were collected in the interface area surrounding cultivated area in Tecomán, Colima region in 2021. **(B)** Total DNA (100 ng) of 12 symptomatic *Galium mexicanum* samples were used for PCR-based molecular detection of GLDV using viral specific primers. (–), negative non template control, MW, molecular weight marker, (+ 1), and (+ 2) plasmidic GLDV-1, and GLDV-2 isolates positive controls, respectively, S, symptomatic plants, AS, asymptomatic plants. The size of expected amplicon is indicated.

In a previous study, we have performed a HTS metagenomic approach in order to identify geminivirus diversity in non-cultivated plants (belonging to 34 families) collected in ecosystems surrounding cultivated areas (agro-ecological interface) located in seven states of the Northern-Pacific Mexico region (Rodríguez-Negrete et al., 2019). The assembly of metagenomic data revealed geminivirus-related DNA signatures (3,00-2,700 bp in length) with ≥ 90% identity with begomoviral sequences present in a genome databank, allowing for the identification of viral molecules at the species level. However, a subset of geminivirus-related signatures showed ≤ 80% identity with reported begomovirus; hence, according to the current begomovirus species demarcation criteria (≤ 89% homology) (Brown et al., 2015), these signatures could be related to novel begomovirus species. A geminivirus-related signature of 465 bp from the Colima state library showed an 80% similarity with SPLCV from China (accession number: MH602265). SPLCV is an OW monopartite begomovirus belonging to a sweepovirus clade, and to date, monopartite begomoviruses have not been reported in Mexico. In order to identify plants harboring the putative begomovirus associated with SPLCV-like signature, a specific primer set, SP-Like-F/SPLike-R (expected amplicon: 232 bp), was used for molecular detection. A total of 49 plants belonging to nine families, *Amaranthaceae* (1), *Asteraceae* (3), *Convolvulaceae* (4), *Cucurbitaceae* (2), *Malvaceae* (28), *Nyctaginaceae* (4), *Rubiaceae* (2), *Solanaceae* (4), and *Vervenacea* (1) from the Colima state sample collection, were individually analyzed by PCR. The expected amplicon (232 bp) was detected in one sample corresponding to the *Galium mexicanum* species belonging to the *Rubiaceae* family (data not shown) collected in Tecomán, Colima, Mexico (coordinates: 18°50′53.07′′ N, 103°49′54.892′′ W) showing leaf distortion and foliar chlorosis symptoms (Figure 1). Afterward, in order to obtain putative full-length begomovirus genomes, circular DNA from a *Galium mexicanum* SPLCV-like signature-positive sample was amplified by rolling circle amplification (RCA), and the resulting concatemers were digested independently with *Bam*HI and *Sac*I, yielding one fragment of 2.7 kb and two fragments of 1.8 and 0.9 kb, and two independent clones of each fragment were fully sequenced. The assembly and sequence comparison showed two putative full-length genomes of 2,656 and 2,658 bp from ∼2.7-kb cloned fragments sharing 91.34% sequence identity between them. Additionally, the alignment of 1.8- and 0.9-kb fragments using full-length genome-1 (2,556 bp) and full-length genome-2 (2,658 bp) sequences as a reference resulted in ∼100% homology with genome-1 of one 1.8-kb fragment and one 0.9-kb fragment. Similarly, the remaining two fragments of 1.8 and 0.9 kb, showed ∼100% homology with genome-2. These data show that *Sac*I fragments (1.8 and 0.9 kb) are split forms of the full-length genome. Nucleotide sequences of both genomes were compared with the begomovirus database of NCBI using the BLASTn algorithm. The 2,656- and 2,658-bp genomes showed 79.88 and 79.2% identity with the DNA-A of the NW begomovirus *Tomato leaf deformation virus* (ToLDeV), isolate PA10-S9 (Accession number: JX501506.1), and *Sida mosaic Bolivia virus* (SiMBoV) (Accession number: KJ742421.1). To identify the putative cognate DNA-B segment, total DNA from *Galium mexicanum* positive sample was used as a template for PCR using two sets of universal degenerate primers BC1-290-for/BV1-470-rev and BC1-290-rev/BV1-310-for (Gregorio-Jorge et al., 2010), yielding negative results with both sets of primers (Supplementary Figure 1), suggesting the presence of a monopartite begomovirus. According to present taxonomic demarcation criteria (≤ 89% for species and ≥ 91% identity for isolates) (Brown et al., 2015), the 2,656- and 2,658-bp genomes represent two isolates of a new begomovirus species, and the name *Galium leaf distortion virus* (GLDV) is proposed. Hereafter, GLDV-1 (for the 2,656-bp genome) and GLDV-2 (for the 2,658-bp genome). Both sequences were submitted in GenBank (GLDV-1 accession number: OL689630, GLDV-2 accession number: OL689631). To validate the obtained data and determinate the geographic distribution of GLDV across agro-ecological interfaces in the Northern Pacific Mexico region, RCA-cloned GLDV-1 and GLDV-2 genome sequences were aligned with the complete metagenomic data from Rodríguez-Negrete et al. (2019) containing the virome present in non-cultivated plants from five agro-regions. Alignment of the GLDV-1 and GLDV-2 genomes against complete metagenomic data yielded a high virus genome assembly coverage (97.06 and 98.3% for GLDV-1 and GLDV-2, respectively) (Supplementary Figure 2) is important to mention that the finding was exclusively from Colima-Nayarit metagenomic data. Finally, to evaluate the current prevalence of *Galium leaf distortion virus* (GLDV) in the original sampling region, 12 samples of *Gallium mexicanum* showing leaf distortion and foliar chlorosis symptoms were collected in Tecomán, Colima in 2021 (Figure 2A) and tested for PCR-based viral molecular detection using *Galium leaf distortion virus* (GLDV)-specific primers (IR GLDV-F/IR GLDV-R), resulting in 8 out of the 12 plants being positive for GLDV infection (Figure 2B). Furthermore, amplicons were sequenced, and the analysis indicated that the isolate GLDV-1 was predominantly present in the samples collected in 2021 (data not shown). Altogether, these data show that *Galium mexicanum* is a reservoir of novel begomovirus species, putatively endemic in the Colima, Mexico agro-region and currently established in ecosystems surrounding cultivated areas.

### Galium Leaf Distortion Virus Displays an NW Begomovirus Genetic Structure and Belongs to an NW Monopartite Clade

**FIGURE 3:**
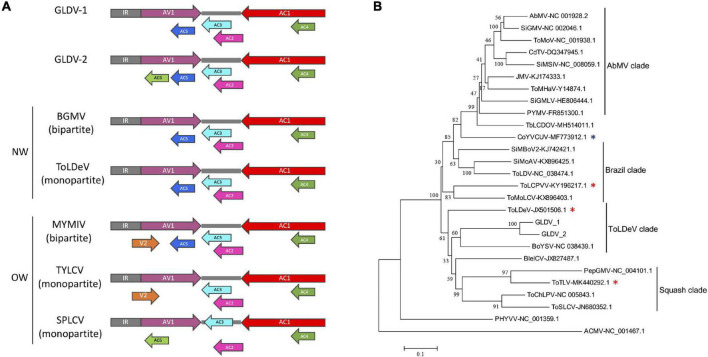
*Galium leaf distortion virus* (GLDV) isolates genomic organization and phylogenetic analysis. **(A)** Comparison of GLDV isolates genomic organization with representative old world (OW) and new world (NW) begomoviruses. Virion and complementary sense open reading frames (ORFs) are indicated with colored arrows, and intergenic regions (IR) are indicated with gray blocks. **(B)** Maximum likelihood (ML) phylogenies were constructed by multiple alignments of complete DNA-A of the two GMV isolates obtained in the present study, and 24 selected DNA-A sequences representative of new world (NW) begomoviruses. ML method in MEGA 7 was based on the Tamura-Nei model and the tree support was tested by bootstrapping with 1,000 replicates. The scale bar represents the genetic distance. The genomic DNA-A sequence of *African cassava mosaic virus* (ACMV), an old world (OW) begomovirus was used as outgroup. The grouping of viruses belonging to the main NW begomoviruses clades including AbMV, Brazil, ToLDeV, and squash is indicated. The NW monopartite begomovirus infecting tomato and non-cultivated plants are indicated with red and blue asterisks, respectively. Accession numbers of viral isolates are shown. Begomovirus acronyms are summarized in Supplementary Table 1.

A comparative analysis of GLDV genomic organization with representative DNA-A segments of NW and OW begomoviruses was performed (Figure 3A). Both GLDV-1 and GLDV-2 displayed six open reading frames (ORFs). For both GLDV isolates, in virion strand sense the AV1/CP gene encoding the capsid protein containing the N-terminal motif PWRsMaGT, which is characteristic of NW begomoviruses is observed, and in the same genomic location, lack of pre-capsid V2 gene which is observed only in bipartite *Mungbean yellow mosaic India virus* (MYMIV) and monopartite *Tomato yellow leaf curl virus* (TYLCV) OW begomoviruses (Khan et al., 2013; Mishra et al., 2020). Moreover, four genes in the complementary sense AC1/Rep, AC2/TrAP, AC3/REn, and AC4 are involved in viral replication/transcription, regulation of host cell cycle, and suppression of plant antiviral responses (Hanley-Bowdoin et al., 2013), and resemble the genomic organization of both bipartite *Bean golden mosaic virus* (BGMV) and the monopartite *Tomato leaf deformation virus* (ToLDeV) NW begomovirus. Additionally, in the complementary strand sense (overlapping with the AV1 gene), both GLDV-1 and GLDV-2 encode an ORF corresponding to the AC5 gene conserved in different NW (BGMV and ToLDeV) and OW *Mungbean yellow mosaic India virus* (MYMIV) begomoviruses. Intriguingly, the GLDV-2 isolate encodes an ORF putatively encoding the AC6 gene in a complementary strand sense partially overlapped with the AV1 gene, which is syntenically conserved in some OW monopartite begomoviruses belonging to the sweepovirus clade including SPLCV. Finally, both GLDV isolates possess an intergenic region (IR) containing the nonanucleotide TAATATTAC conserved in the *Geminiviridae* family located in a predicted stem-loop structure, which marks the origin of geminiviral DNA replication (Lazarowitz, 1987). On the other hand, a phylogenetic tree based on multiple alignments of complete nucleotide sequences of both GLDV isolates and selected begomoviruses belonging to main clades present in the American continent (*Abutilon mosaic virus* (AbMV) Brazil is a clade, *Tomato leaf deformation virus* (ToLDeV), and squash clades) available in the GenBank database showed that GLDV-1 and GLDV-2 are placed in the ToLDeV clade together with *Boerhavia yellow spot virus* (BoYSV) (Figure 3B). ToLDeV is a tomato-infecting NW monopartite begomovirus, whereas for BoYSV only DNA-A has been identified. Additionally, a recombination analysis using both the isolates of GLDV and NW begomovirus DNA-A genomes available in the GenBank database was performed. No recombination events were detected, with more than two methods yielding not significant results (data not shown) suggesting that GLDV parental sequences have not been currently discovered. Together, these results indicated that GLDV possesses a DNA-A-like NW begomovirus genetic structure that is phylogenetically related to NW monopartite begomoviruses.

### Galium Leaf Distortion Virus-1 and Galium Leaf Distortion Virus-2 Possess Divergent Genomic Regions

In order to characterize at the molecular level, the GLDV-1 and GLDV-2 isolate genomes, comparative nucleotide, and amino acid analyses were performed (Supplementary Figures 3, 4 and Supplementary Table 2). GLDV-1 (2,656 bp) and GLDV-2 (2,658 pb) share 91.34% of nucleotide identity with 222 different nucleotides. GLDV-1 and GLDV-2 possess an intergenic region (IR) of 281 and 283 nt, respectively, with 91.59% identity. Interestingly, a comparison of the left IR and the right IR (relative to stem-loop structure) showed an existing divergence in the left IR (where viral DNA replication regulatory elements are located) compared with the right IR, with 85 vs. 92.2% nucleotide identity, respectively. Coding regions of the AV1 and AC1 genes were highly conserved among GLDV isolates (> 92 and > 93% of nucleotide and amino acid identity for the AV1 and AC1 genes, respectively), followed by the AC4 gene (94.57 and 89.41% for nucleotide and amino acid identity, respectively), whereas AC2, AC3, and AC5 were more divergent, showing amino acid similarities < 89%. Additionally, the comparison performed with the top six most similar genomes [including *Tomato leaf deformation virus* (ToLDeV) and *Boerhavia yellow spot virus* (BoYSV), belonging to the same GLDV clade] confirmed the high divergence of GLDV isolates compared with their closest relatives from Latin America (Mexico, Peru, Brazil, and Argentina) (Supplementary Table 2). To identify putative molecular/functional features resulting from genomic differences among the GLDV isolates, a more detailed analysis of divergent genomic regions (IRs, AC2, AC3, and AC5) is necessary.

### Galium Leaf Distortion Virus-1 and Galium Leaf Distortion Virus-2 Display a Different Iteron Structure and Conserve Replication-Associated Molecular Vestiges of the Sweepovirus Lineage

**FIGURE 4:**
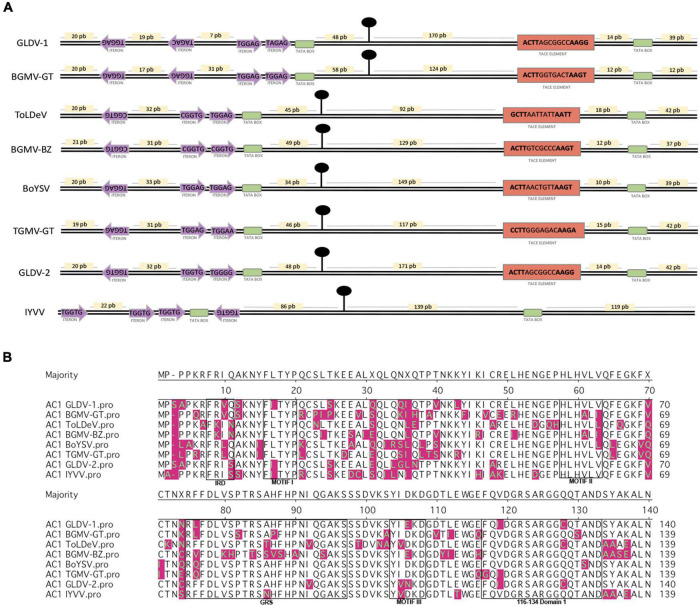
*Galium leaf distortion virus* (GLDV) isolates replication/transcription regulatory elements analysis. **(A)** Comparative analysis of regulatory elements present in intergenic region (IR) of DNA-A components of GLDV isolates and selected NW begomoviruses. Arrangement and sequence of replication-associated iterated elements (iterons), and TATA-associated composite element (TACE), are shown in violet arrows and orange rectangles, respectively. For TACE element, dyadic four bases element is shown in bold. Replication origin and TATA-boxes, are indicated by black circles and green boxes, respectively. Nucleotide distances between regulatory elements is shown in yellow boxes. **(B)** Comparative analysis of N-terminal region (140 aa) of AC1 proteins of GLDV isolates and selected NW begomoviruses. Iteron-related domain (IRD), Motif I, II, III, and GRS, and 116–134 motifs are indicated. Differential amino acid residues compared with sequence consensus are indicated in magenta. Begomovirus accession numbers and acronyms are summarized in Supplementary Table 1.

The IRs of dicot-infecting geminiviruses contain regulatory elements involved in viral DNA replication and gene transcription regulation and whose arrangement and consensus sequences are conserved in different geminivirus lineages from the OW and NW or the Western and Eastern hemispheres. The left IR (relative to stem-loop structure) contains replication-associated iterated elements (iterons) that are virus-specific, whereas the right IR contains transcriptional regulatory elements of the AV1/CP gene (i.e., TATA box and conservative late element, CLE) (Argüello-Astorga et al., 1994). An analysis of IR regulatory elements of both GLDV isolates and two other members of its clade (ToLDeV and BoYSV) was performed (Figure 4A). GLDV-1 left IR sequence inspection revealed that this isolate shows an arrangement of two directed TGGAG/TAGAG and two inverted TAGAC/TGGAG iteron cores similar to the *Fabaceae* family infecting NW bipartite begomovirus BGMV-GT (Guatemala strain), which possess the same iteron arrangement with TGGAG consensus. Left IRs of ToLDeV, BoYSV, and GLDV-2 display an arrangement of two directed and one inverted iteron conserved among different lineages of NW begomoviruses. ToLDeV possesses direct CGGTG/TGGAG and reverses CGGTG iteron cores similar to NW bipartite begomovirus *Bean golden mosaic virus* (BGMV-BZ, Brazil strain) infecting the *Fabaceae* family with CGGTG consensus, whereas BoYSV shows direct TGGAG/TGGAG and reverse TGGAG iteron cores similar to NW bipartite begomovirus *Tomato golden mosaic virus* Guatemala strain TGMV-GT infecting the *Solanaceae* family with TGGAG consensus. Intriguingly, in spite of GLDV-2 showing an iteron arrangement conserved among NW begomoviruses, the direct TGGTG/TGGGG and reverse TGGTG iteron sequences are shared with OW begomovirus *Ipomea yellow vein virus* (IYVV) belonging to the sweepovirus clade and infecting the *Convolvulaceae* family, showing OW begomovirus iteron arrangement (three directed iteron up-stream TATA box and one iteron down-stream TATA box) with sequence consensus TGGTG (Figure 4A). On the other hand, the right IRs of all NW begomoviruses analyzed possess a TATA-associated composite element (TACE) (Figure 4A) similar to the conservative late element (CLE) involved in AV1/CP gene expression driven by AC2 transactivation. The TACE element is characterized by a hypervariable seven-base core flanked by a dyadic four-base element with ACTT-(N7)-AAGT consensus closely associated with a TATA box, and is a hallmark of NW begomoviruses (Cantú-Iris et al., 2019). The geminivirus-encoded replication initiator protein AC1/Rep binds in a sequence-specific fashion to iterons through the iteron-related domain (IRD) in order to initiate viral replication (Argüello-Astorga and Ruiz-Medrano, 2001). A comparative analysis of the N-terminal region (140 AAs) of corresponding AC1 proteins showed that GLDV-1 presents an IRD motif, FRVQ, shared with the *Bean golden mosaic virus* Guatemala strain (BGMV-GT), *Tomato leaf deformation virus*(ToLDeV), and *Boerhavia yellow spot virus* (BoYSV) FKIN and FRLQ IRD motifs shared with BGMV-BZ and TGMV-GT, respectively, whereas GLDV-2 shows the IRD FRIS motif shared with IYVV (Figure 4B), which is in agreement with the iteron sequence analysis. In addition to the IRD motif, the Rep N terminus contains three conserved motifs: Motif I, which is required for specific dsDNA binding, Motif II, which is a metal-binding site necessary for DNA cleavage, and Motif III, which is the catalytic site for DNA cleavage, and two domains: the GRS domain, which is required for infection and viral genome replication (Nash et al., 2011), and the 116-134 domain, which is conserved in OW and NW begomoviruses and has unknown functions (Torres-Herrera et al., 2019). Motif I is the most conserved in the *Geminiviridae* family (Nash et al., 2011), as revealed by the N terminus of the AC1 protein alignment of representative members of the fourteen genera of the *Geminiviridae* family showing the Motif I consensus FLTYP in 10 genera including OW and NW *Begomovirus*, FLTYS in two genera (*Becurtovirus* and *Eragrovirus*), FLTFP in two genera (*Opunvirus* and *Mulcrilevirus*), and interestingly, OW begomoviruses belonging to the sweepovirus clade, SPLCV, IYVV, and SPLCCNV, and both GLDV isolates showed FITYP consensus including the substitution of Isoleucine (I) instead Leucine (L) in the second position of Motif I FLTYP conserved in begomovirus genera (Supplementary Figure 5 and Figure 4B). The remaining Motifs, II and III, and domains GRS and 116-134 showed no significant differences (Figure 4B). Altogether, the GLDV-1 and GLDV-2 IRs and AC1 motif/domain analysis suggest that each viral isolate could possess different replicative adaptations and that both molecules conserve molecular vestiges of the sweepovirus lineage.

### Divergence of Galium Leaf Distortion Virus-1 and Galium Leaf Distortion Virus-2 Genes (AC2, AC3, and AC5) Is Associated With Putative Different Functional Features

**FIGURE 5:**
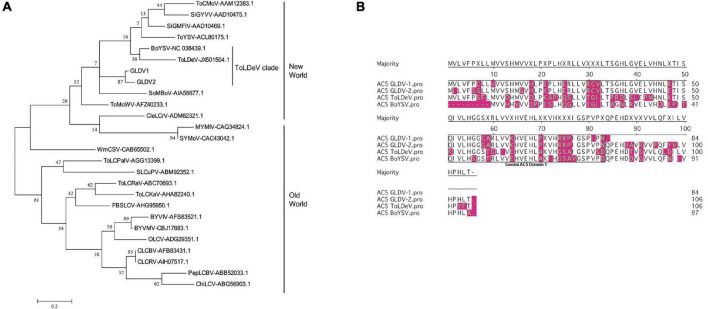
Phylogenetic and amino acid analysis of *Galium leaf distortion virus* (GLDV) isolates AC5 proteins. **(A)** Maximum likelihood (ML) unrooted phylogenetic tree based on nucleotide sequences of AC5 coding regions. ML phylogenies were constructed by multiple alignments of GLDV isolates open reading frames (ORFs) of AC5 gene, and 24 selected AC5 sequences representative of new world (NW) and old world (OW) begomoviruses. ML method in MEGA 7 was based on the Tamura-Nei model and the tree support was tested by bootstrapping with 1,000 replicates. The scale bar represents the genetic distance. The division of NW, OW, and ToLDeV clades is indicated. Accession numbers of viral isolates are shown. Begomovirus acronyms are summarized in Supplementary Table 1. **(B)** Alignment of AC5 amino acid sequences of viruses belonging to ToLDeV clade. Differential amino acid residues are highlighted in magenta. Gemini AC5 Domain 1 was identified using the conserved domain database (CDD).

The product of the AC2/TrAP gene is a ∼15-kDa protein conserved among all begomoviruses that play a central role in the viral replication cycle, transcriptional regulation of late viral genes, and suppression of host defense including gene silencing (Guerrero et al., 2020). The amino acid alignment of GLDV-1 and GLDV-2 AC2 proteins showed that both proteins present conserved structural features: basic region, zinc finger domain (containing conserved cysteine and histidine residues), acidic region, and minimal activation domain. The basic region is identical between both GLDV isolates, whereas point changes are observed in the remaining regions (Supplementary Figure 6A). Previous cellular localization experiments have shown that the non-phosphorylated form of AC2 is found in the host nucleus and cytoplasm, whereas phosphorylated AC2 is only found in the nucleus, suggesting that the function of viral proteins is regulated by this posttranslational modification (Hartitz et al., 1999; Wang et al., 2003). A prediction of putative phosphorylation sites (Serine and Threonine residues) of AC2 proteins of both GLDV isolates (using the NetPhos 3.1 server^4^ showed a different phosphorylation pattern between AC2 proteins (Supplementary Figure 6B). For both GLDV isolates, a total of 13 putative phosphorylation sites were predicted; however, the phosphorylation pattern showed some differences. For GLDV-1 and GLDV-2, serines (Ss) 5, 6, 7, 57, 70, 105, and 108, and threonine (Ts) 8, 21, 94, and 106 were predicted as commonly phosphorylated sites. Whereas Ss 58, 88, 100, 120, and 121, and T 48 were predicted only for GLDV-1, and Ss 48 and 119, and T 52, 79, 80, and 117 were only predicted for the GLDV-2 isolate (Supplementary Figure 6B). These data suggested that GLDV-1 and GLDV-2 AC2 proteins could be targeted differentially at the posttranslational level, also resulting in a distinct spatial/temporal regulation. Dicot-infecting geminiviruses encode a conserved replication enhancer protein (AC3/REn) of ∼15.5 kDa, which is required for viral DNA replication throughout its interaction with the AC1 protein. AC3 also homo-oligomerizes and interacts with host protein retinoblastoma-related protein (pRBR) (Hanley-Bowdoin et al., 2004) and proliferating cell nuclear antigen (PCNA) (Castillo et al., 2003), which are involved in cellular cycle regulation. Based on yeast two-hybrid (Y2H) protein-protein interaction assays, AC3 interactor binding sites have been mapped for *Tomato yellow leaf curl virus* (TYLCV) and *Tomato golden mosaic virus* (TGMV) AC3 proteins (Settlage et al., 2005) (Supplementary Figure 7A). Additionally, directed mutagenesis studies showed the existence of amino acid residues involved in AC3 interactors’ binding efficiency, affecting their replication enhancement activity by potential protein structural changes (Settlage et al., 2005). A GLDV isolate AC3 protein secondary structure analysis showed that both proteins share different regions of helices and sheets in N-terminal and central regions; however, a differential sheet structure is observed in the C-terminal region between both isolates (Supplementary Figure 7B). Furthermore, the merging of predicted tertiary structures showed different spatial folding of both AC3 proteins (Supplementary Figure 7C). The AC5 gene present in several bipartite and monopartite begomoviruses has been described as a virulence factor and a suppressor of gene silencing at posttranscriptional (PTGS) and transcriptional (TGS) levels. An AC5 gene is highly divergent and varies in length among begomoviruses divided into two main groups: proteins ranging from 83 to 134 AAs and other groups with AAs ranging between 172 and 225. Furthermore, AC5 genes display two different genetic structures with one or two conserved domains, namely, the Gemini AC5-1 and Gemini AC5-2 domains (Li et al., 2015). A phylogenetic tree based on AC5 nucleotide sequences from GLDV isolates and selected OW and NW begomoviruses showed that both GLDV-1 and GLDV-2 are grouped with *Tomato leaf deformation virus* (ToLDeV) and *Boerharvia yellow spot virus* (BoYSV) (ToLDeV clade), and with other NW begomoviruses according to those with full-length genome-based phylogeny (Figure 5A). Additionally, the amino acid alignment of AC5 proteins of members of the ToLDeV clade showed that AC5 proteins are encoded by 84 amino acids (AAs) for GLDV-1, 106 AAs for GLDV-2 and ToLDeV, and 97 AAs for BoYSV (Figure 5B). The difference in AC5 protein length among GLDV isolates is caused by a premature stop codon in GLDV-1 AC5 codon 85. Despite the difference in length of AC5 proteins among GLDV isolates, ToLDeV, and BoYSV, all proteins only contain the conserved Gemini AC5-1 domain (Figure 5B). The biological relevance of predicted differential molecular and structural GLDV AC2, AC3, and AC5 gene features needs to be experimentally studied.

### Infectivity Assays Corroborate the Monopartite Nature of GLDV

**FIGURE 6:**
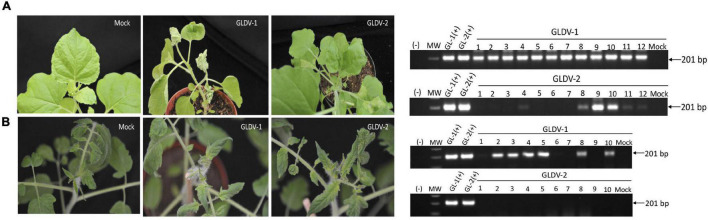
*Galium leaf distortion virus* (GLDV) infectivity assay. *Nicotiana benthamiana* and *Solanum lycopersycum* plants were inoculated with GLDV-1 or GLDV-2 agroinfectious clones, and symptoms documented at 21 dpi (**A,B**, right panel, respectively). Total DNA (100 ng) of *N. benthamiana* (*n* = 12) and *S. lycopersicum* (*n* = 10) plants inoculated with GLDV-1 or GLDV-2 agroinfectious clones was used as template for PCR molecular detection using GLDV specific primers (**A,B**, left panel, respectively). (–), negative non template control, MW, molecular weight marker, GL-1 (+), and GL-2 (+), plasmidic positive controls of GLDV-1, and GLDV-2, respectively, Mock, control plant inculcated with agrobacterium harboring empty binary vector. The size of expected amplicon is indicated.

Molecular and phylogenetic analyses suggest that GLDV is an NW monopartite begomovirus. To test the monopartite nature of GLDV isolates at the biological level, hemidimeric clones obtained from corresponding viral DNA-A segments were agroinoculated in the experimental host *Nicotiana benthamiana* and tomato (*Solanum lycopersicum*) in three independent experiments. *Nicotiana benthamiana* plants inoculated with GLDV-1 displayed symptoms such as stunting and severe leaf curling, and were PCR-positive (100%, 12/12 plants), whereas plants inoculated with the GLDV-2 isolate remained predominately asymptomatic 21 dpi and, nonetheless, showed 50% infectivity by PCR molecular detection (6/12 plants) (Figure 6A). On the other hand, inoculation of both GLDV isolates in tomato plants resulted in asymptomatic phenotype 21 dpi. However, for GLDV-1, it was PCR-detected, with infectivity of 60 (6/10 plants) and 0% (0/10 plants) for the GLDV-2 isolate (Figure 6B). These results corroborate the monopartite nature of GLDV isolates.

## Discussion

Viruses are the largest group of emerging pathogens, and members of the *Geminiviridae* family with single-stranded DNA genomes, particularly *Begomovirus* genera, are the major group of emerging plant viruses (García-Arenal and Zerbini, 2019). Viral plant disease emergence is the final result of alteration in multitrophic interactions (plant-virus-insect vector) by abiotic/biotic and anthropogenic factors. From the early 1900s to date, factors such as increasing population worldwide, global warming, the dependence of monocultures, global movement of contaminated germplasm, and re-distribution of insect vectors, have facilitated the emergence of geminivirus diseases causing devastating yield loss in many, such as bean, cassava, cotton, cucurbits, and tomato in tropical and subtropical regions of the world (Accotto et al., 2000; Briddon and Markham, 2000; Brown et al., 2002; Canto et al., 2009; Patil and Fauquet, 2009). Traditionally, the epidemiology of geminivirus diseases has been focused primarily on crop plants. Nevertheless, knowledge of geminivirus infection in non-cultivated plants especially in the interface between crops and natural ecosystems is necessary to provide a complete landscape of plant virome (García-Arenal and Zerbini, 2019). Recently, HTS coupled to metagenomic and ecogenomic approaches has provided a powerful tool for detecting known viruses identifying novel viral species in a high-resolution fashion, allowing for identification of genetic variations in viral populations and providing the necessary input to understand viral genome evolution, epidemiology, and ecology of disease emergence (Barba et al., 2014; Massart et al., 2014; Wu et al., 2015). In this study, two isolates of a novel NW monopartite begomovirus species tentatively named *Galium leaf disstortion virus* (GLDV) according to the current ICTV demarcation criteria were discovered by data mining of our previous reported metagenomic data (Rodríguez-Negrete et al., 2019) and characterized at the molecular, phylogenetic, and biological levels. The PCR molecular screening of an SPLCV-like signature from the Colima state NGS library, followed by RCA-based full-genome begomovirus isolation, showed that Mexican endemic non-cultivated plant *Galium mexicanum* (*Rubiaceae* family) is the reservoir of GLDV. Non-cultivated plants belonging to the *Rubiaceae* family, such as *Galium mexicanum*, are ranked as the third most important group of plants used in traditional ethno-medicine in Mexico (Andrade-Cetto, 2009), highlighting the importance of this group of plants acting not only as reservoirs but also hosts of viral species. In addition, these species are widespread in the region and are herbicide-tolerant, which could increase the potential risk as a reservoir plant. The geographic distribution of GLDV was restricted to the Colima state region according to the georeferenced mapping of total metagenomic reads from five agro-regions of Northern Pacific Mexico (Rodríguez-Negrete et al., 2019) using the complete genome of both GLDV isolates as reference. Additionally, a prevalence analysis by means of molecular detection of GLDV in symptomatic *Galium mexicanum* plants collected in the same region (Colima state) in 2021 showed that GLDV is currently established in these ecosystems surrounding cultivated areas, warning about a possible viral spillover to crops. Interestingly, the sequence of the amplicons obtained with specific primers is related to the GLDV-1 isolate, suggesting better fitness in such an environment. Recently, georeferenced metagenomics, (geometagenomics), has precisely linked individual sequence reads to plant hosts from which they were obtained and spatial arrangement of these hosts, enabling to study impacts of host diversity, host spatial variations, and environmental conditions on plant virus diversity and prevalence (Claverie et al., 2018). The molecular analysis of GLDV isolates sequences, showed that both GLDV-1, and GLDV-2 genomes display the genetic arrangement typical of DNA-A segment of NW begomoviruses: In the virion strand sense AV1/CP, and AC5 (conserved in some NW begomoviruses) genes, four genes in the complementary sense AC1/Rep, AC2/TrAP, AC3/REn, and AC4, and an Intergenic Region (IR) containing the viral replication-associated non-anucleotide TAATATTAC element conserved in *Geminiviridae* family (Lazarowitz, 1987; Hanley-Bowdoin et al., 2013). Intriguingly, the GLDV-2 isolate encodes an ORF putatively encoding AC6 in a complementary strand sense partially overlapped with the AV1 gene, which is syntenically conserved in some OW monopartite begomoviruses belonging to the sweepovirus clade. Recently, it has been described that additional hypothetical ORFs encoded by the *Tomato yellow leaf curl virus* (TYLCV) genome are expressed and play a role during viral infection, implying that the repertoire of geminiviral proteins can be expanded (Gong et al., 2021). A phylogenetic analysis using representative members of the main begomovirus American clades for tree construction grouped both GLDV isolates in the clade of the monopartite NW begomovirus, *Tomato leaf deformation virus* (ToLDeV). The phylogenic relationship of GLDV with a monopartite begomovirus and lack of cognate DNA-B (tested by PCR molecular detection using universal primers specific for DNA-B) suggested that GLDV possesses a monopartite nature. Interestingly, a recombination analysis using both isolates of GLDV and all reported sequences of NW begomovirus DNA-A genomes did not render significant results, suggesting that GLDV parental sequences have not been currently discovered. A comparative gene-by-gene analysis of GLDV sequences showed that both isolates share high homology in AV1 and AC1 coding regions (> 92 and > 93% of nucleotide and amino acid identity, respectively), in agreement with the conservation of these genes in the *Geminiviridae* family, followed by the AC4 gene (94.57 and 89.41% for nucleotide and amino acid identity, respectively). On the other hand, divergent regions were observed, including the left IR (85% nucleotide identity), AC2, AC3, and AC5 (< 89% amino acid similarities), and analyzed more in detail. The IRs of geminiviruses contain transcription and replication-associated elements, whose arrangement and consensus are conserved in OW and NW linages. The left IR (relative to stem-loop structure) contains replication-associated iterated elements (iterons) that are virus-specific, whereas the right IR contains transcriptional regulatory elements of the AV1/CP gene (i.e., TATA box and conservative late element, CLE) (Argüello-Astorga et al., 1994). The left IR sequence of both GLDV-1 and GLDV-2 showed an arrangement of iterons conserved in NW begomovirus linages, infecting the *Fabaceae* and *Solanaceae* families, respectively (two direct and two inverted iterons for the GLDV-1 present in a *Bean golden mosaic virus* Guatemala isolate (BGMV-GT), and two direct and one inverted iterons for the GLDV-2 present in a *Tomato golden mosaic virus* Guatemala isolate (TGMV-GT)). However, a GLDV-1 iteron consensus (TGGAG) is shared with NW begomovirus BGMV-GT, and, intriguingly, a GLDV-2 iteron consensus (TGGTG) is shared with OW begomovirus Ipomea yellow vein virus (IYVV) belonging to the sweepovirus clade and infecting the *Convolvulaceae* family. Sweepoviruses are monopartite and infect Ipomoea species worldwide, and they phylogenetically belong to a branch distinct from the OW and NW begomovirus linages; this branch seems to represent one of the earliest points of divergence in *Begomovirus* genera (Bi et al., 2017), suggesting that viral species such as GLDV (showing sweepovirus vestiges) could be part of this evolutive transition. On the other hand, the right IR of both GLDV isolates possess a TATA-associated composite element (TACE) similar to the conservative late element (CLE) characterized by a hypervariable seven-base core flanked by a dyadic four-base element with an ACTT-(N7)-AAGT consensus, which is a hallmark of NW begomoviruses (Cantú-Iris et al., 2019). To initiate viral replication, replication initiator protein AC1/Rep binds to iterons in a sequence-specific manner through the iteron-related domain (IRD) located in the N-terminal region of the protein (Argüello-Astorga and Ruiz-Medrano, 2001). GLDV-1 presents an IRD motif, FRVQ, shared with BGMV-GT, whereas GLDV-2 shows the IRD FRIS motif shared with IYVV, in agreement with the iteron sequence analysis. Additional to the IRD motif, the Rep N terminus contains five more motifs. Motif I is required for specific dsDNA binding and is the most conserved in the *Geminiviridae* family (Nash et al., 2011), showing the consensus FLTYP in 10 of the fourteen genera including OW and NW begomoviruses. Interestingly, both GLDV isolates show substitution of isoleucine (I) instead of leucine (L) in the second position of Motif I (FITYP), which is only observed in some begomovirus members of the sweepovirus clade. The remaining Motifs II (a metal-binding site necessary for DNA cleavage) and III (the catalytic site for DNA cleavage), GRS (required for infection and viral genome replication) (Nash et al., 2011), and 116-134 (conserved in OW and NW begomoviruses with unknown functions) (Torres-Herrera et al., 2019) did not show significant differences compared with other NW begomoviruses. These results reinforce the notion of the presence of vestigial molecular features in GLDV genomes and could suggest the presence of putative different replicative adaptations (i.e., host range) of viral isolates. For divergent coding regions between the GLDV isolates, molecular comparative analysis was performed. The amino acid alignment of GLDV-1 and GLDV-2 AC2/TrAP proteins showed that both proteins present conserved structural features such as basic region, zinc finger domain (containing conserved cysteine and histidine residues), acidic region, and minimal activation domain (Hartitz et al., 1999). However, the basic region is identical between both GLDV isolates and point changes observed in the remaining regions are correlated with predicted changes in phosphorylation patterns of serine (S) and threonine (T) residues. The AC2 protein is an *in trans* transcriptional regulator and gene-silencing suppressor (Guerrero et al., 2020), and it has been shown that the phosphorylation pattern (of S and T residues) signals the cellular protein localization between the cytoplasm and the nucleus modulating AC2 functions (Hartitz et al., 1999; Wang et al., 2003). *In vitro* phosphorylation of GLDV-1 and GLDV-2 AC2 proteins, followed by cellular localization and protein functional analysis (transactivation and gene silencing suppression) will be necessary to validate the function of predicted different phosphorylation patterns. The alignment of GLDV-1 and GLDV-2 AC3/REn proteins, followed by secondary and tertiary structure prediction, showed that both proteins share different regions of helices and sheets in the N-terminal and central regions. However, a different sheet structure is observed in the C-terminal region between both isolates, and this change is correlated with changes in the spatial tertiary structure of proteins. AC3 is a viral replication enhancer, and its function is controlled by direct protein-protein interactions with host factors including retinoblastoma-related protein (pRBR) and proliferating cell nuclear antigen (PCNA), and viral protein autointeractions between AC1 and AC3 gene products (Castillo et al., 2003; Hanley-Bowdoin et al., 2004). Directed mutagenesis and heterologous protein-protein interaction assays (i.e., yeast two hybrid, Y2H) will be helpful in elucidating AC3 GLDV isolates feature. For the AC5 gene, a phylogenetic tree based on AC5 nucleotide sequences from GLDV isolates and selected OW and NW begomoviruses showed that both GLDV-1 and GLDV-2 have been located in the *Tomato leaf deformation virus* (ToLDeV) clade and with other NW begomoviruses according to those with full-length genome-based phylogeny. Additionally, the amino acid alignment of AC5 proteins of members of the ToLDeV clade showed that AC5 proteins are encoded by 84 amino acids (AAs) for GLDV-1 and 106 AAs for GLDV-2, and contains the conserved Gemini AC5-1 domain. The AC5 gene is conserved in different NW and OW begomoviruses ranging from 83 to 225 AAs and displaying one or two domains (Gemini AC5-1 and Gemini-AC5-2), and has been described as a pathogenicity determinant and gene silencing suppressor. However, in some cases, mutagenic analyses have shown that this gene is a cryptic gene that lacks functionality (Li et al., 2015) and remains to be tested at the experimental level. Finally, to test the molecular- and phylogenetic-based predictions of the monopartite nature of GLDV, infectivity assays were performed on *Nicotiana benthamiana* and tomato (*Solanum lycopersicum*) plants using GLDV-1 and GLDV-2 agroinfectious clones and analyzed 21 dpi. In *Nicotiana benthamiana*, GLDV-1 induced strong symptoms such as stunting and severe curling in all inoculated plants, which remained present until the late infection stage (35 dpi), whereas GLDV-2 inoculation resulted in predominantly an asymptomatic phenotype, and only an initial step of infection in several plants a minor symptom was observed. On the other hand, inoculation of both GLDV isolates in tomato plants resulted in an asymptomatic phenotype 21 dpi. The infectivity efficiency determined by PCR molecular detection showed that GLDV-1 presents 100 and 60% of infectivity in *Nicotiana benthamiana* and tomato, respectively, and that GLDV-2 presents 50 and 0% infectivity in *Nicotiana benthamiana* and tomato, respectively. These results corroborate the monopartite nature of GLDV and the differential infection capability of viral isolates previously predicted at the molecular level. In spite of both GLDV isolates displaying an iteron arrangement conserved in NW begomovirus linages, their iteron consensus is divergent. A GLDV-1 iteron consensus (TGGAG) is shared with NW begomovirus, whereas a GLDV-2 iteron consensus (TGGTG) is shared with OW begomovirus belonging to the sweepovirus clade. Thus, it is possible that differences in pathogenicity and virulence between GLDV isolates (at least in the host tested in this study) could be related to specific replicative adaptations influenced by Rep binding affinity to corresponding iterons and/or with host proviral factors. However, we cannot rule out a putative individual or the combinatorial role of divergent genes (C2, C3, and C5) in viral replication and evasion of host defense by gene silencing suppression or other molecular mechanisms. It is canonically assumed that new world (NW) begomoviruses are bipartite (Brown et al., 2015). However, indigenous NW monopartite begomoviruses infecting tomato have been described: *Tomato leaf deformation virus* (ToLDeV) in Ecuador and Peru (Melgarejo et al., 2013; Sánchez-Campos et al., 2013), *Tomato mottle leaf curl virus* (ToMoLCV) and *Tomato leaf curl purple vein virus* in Brazil (Vu et al., 2015; Macedo et al., 2018), and *Tomato twisted leaf virus* (ToTLV) in Venezuela (Romay et al., 2019). Recently, *Corchorus yellow vein Cuba virus* (CoYVCUV) infecting the non-cultivated plant *Corchorus siliquosus* (family *Malvaceae*) in Cuba (Fiallo-Olivé and Navas-Castillo, 2020) was reported. In the present study, we report the first description of an NW monoportite begmomovirus species in Mexico, and the second report of a monopartite begomovirus infecting a non-cultivated plant in the American continent. Our data revealed additional complexity in monopartite begomovirus genetics and geographic distribution, and highlight the importance of metagenomic approaches in understanding global virome ecology.

## Data Availability Statement

The datasets presented in this study can be found in online repositories. The names of the repository/repositories and accession number(s) can be found below: https://www.ncbi.nlm.nih.gov/genbank/, OL689630; https://www.ncbi.nlm.nih.gov/genbank/, OL689631.

## Author Contributions

EG-R, ER-N, JM-L, and NL-L: conceptualization. EG-R, ER-N, and JM-L: formal analysis, methodology, and writing (original draft). JM-L: funding acquisition and resources. EG-R and ER-N: investigation. ER-N, JM-L, NL-L, and EA-C: supervision. EG-R, ER-N, JM-L, NL-L, and EA-C: writing (review and editing). All authors have read and agreed to the published version of the manuscript.

## Conflict of Interest

The authors declare that the research was conducted in the absence of any commercial or financial relationships that could be construed as a potential conflict of interest.

## Publisher’s Note

All claims expressed in this article are solely those of the authors and do not necessarily represent those of their affiliated organizations, or those of the publisher, the editors and the reviewers. Any product that may be evaluated in this article, or claim that may be made by its manufacturer, is not guaranteed or endorsed by the publisher.

## Footnotes

1
https://galaxy.pasteur.fr/datasets/edit?dataset_id=efa443e64911a5f1

2
https://bioviz.org/

3
https://www.ebi.ac.uk/Tools/msa/clustalo/

4
http://www.cbs.dtu.dk/services/NetPhos/
