# Supplementary material for: From Metagenomics to Discovery of New Viral Species: Galium Leaf Distortion Virus, a Monopartite Begomovirus Endemic in Mexico
Source: Front Microbiol. 2022 Apr 25;13:843035. doi: 10.3389/fmicb.2022.843035 (PMC9083202; doi:10.3389/fmicb.2022.843035)
Supplement: Supplementary file 1 [file Data_Sheet_1.pdf]

frontiers

Supplementary Material

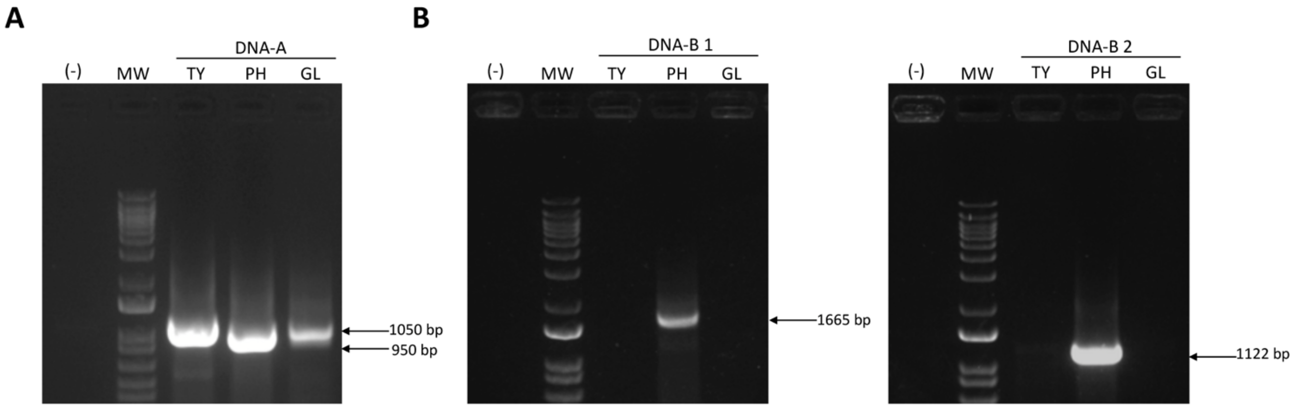

Supplementary Figure 1. **Molecular detection of begomoviruses using Universal primers**. Total DNA (100 ng) of tomato plants infected with TYLCV (TY), or PHYVV (PH), and *Galium mexicanum* infected with GLDV (GL), was used as PCR template. **A)** Detection of DNA-A segment of begomoviruses using universal primers set DGR-Sar/CP-70 previously described (Mauricio-Castillo et al., 2007). **B)** Detection of DNA-B segment of begomovirus using universal primers set BC1-290-for/BV1-470-rev (DNA-B 1), and BV1-310-for/BC1-290-rev (DNA-B 2) (left and right panel, respectively), previously described (Gregorio-Jorge et al., 2010). (-), negative non template control, MW, molecular weight marker. The size of expected amplicons is indicated.

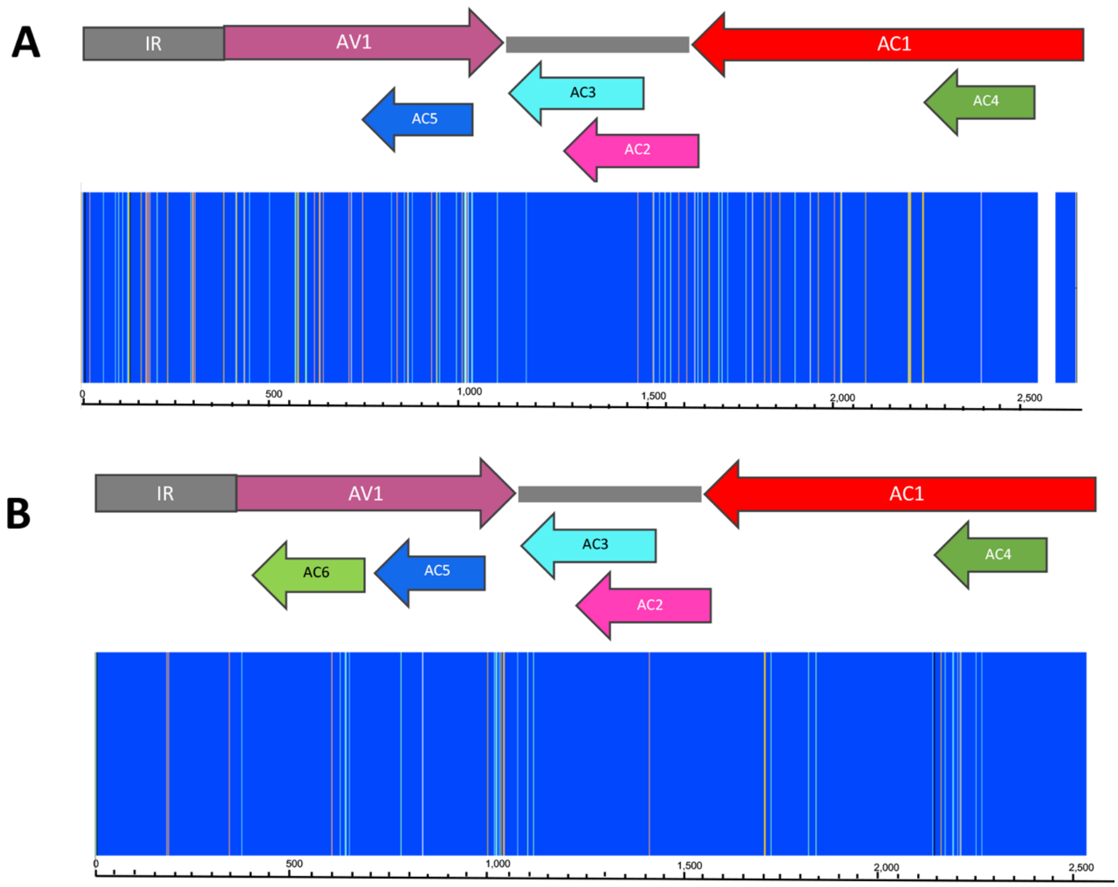

Supplementary Figure 2. ***In silico*** **reference-based** ***Galium leaf distortion virus*** **(GLDV) genome reconstruction**. The complete HTS reads from Rodríguez-Negrete et al., (2019) metagenomic data were mapped using GLDV genomes as reference using Bowtie2 tool in the Galaxy server. Collapsed aligned sequences are shown under reference map for GLDV-1 (**A**), and GLDV-2 (**B**). Dark blue bars represent the assembled regions with 100 % of coincidence, whereas cyan, light blue, pink, and orange bars represent the non-coinciding adenine, thymine, cytosine, and guanine nucleotides, respectively. A scale bar of viral genomic positions is shown.

Supplementary Figure 3

Intergenic Region (nucleotide)

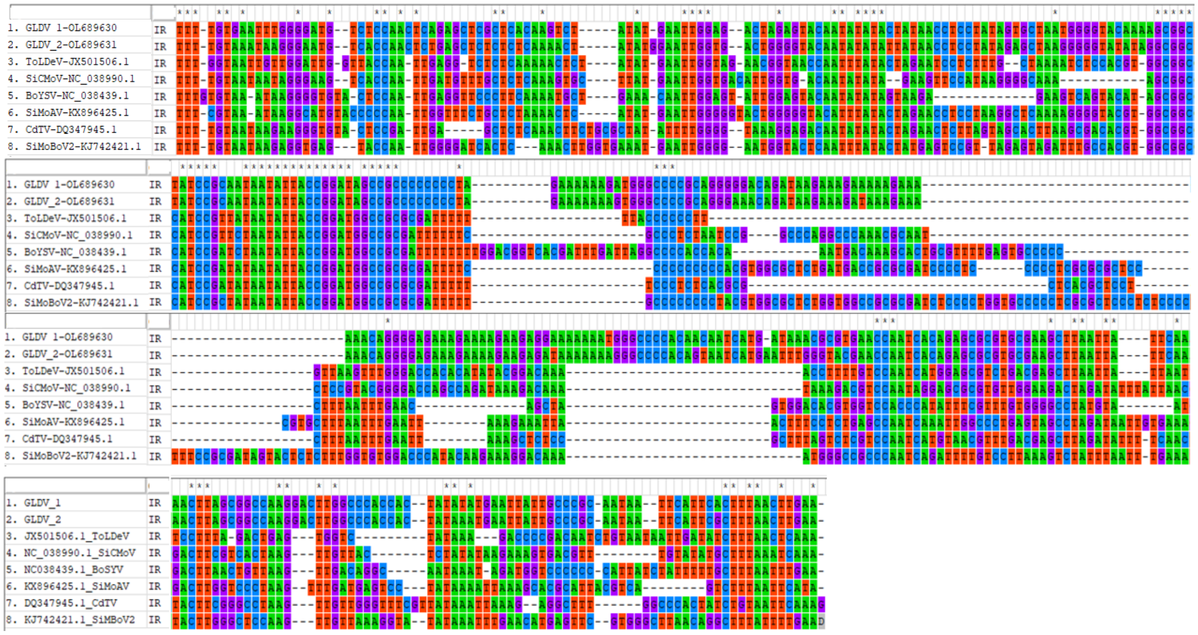

AV1 (nucleotide)

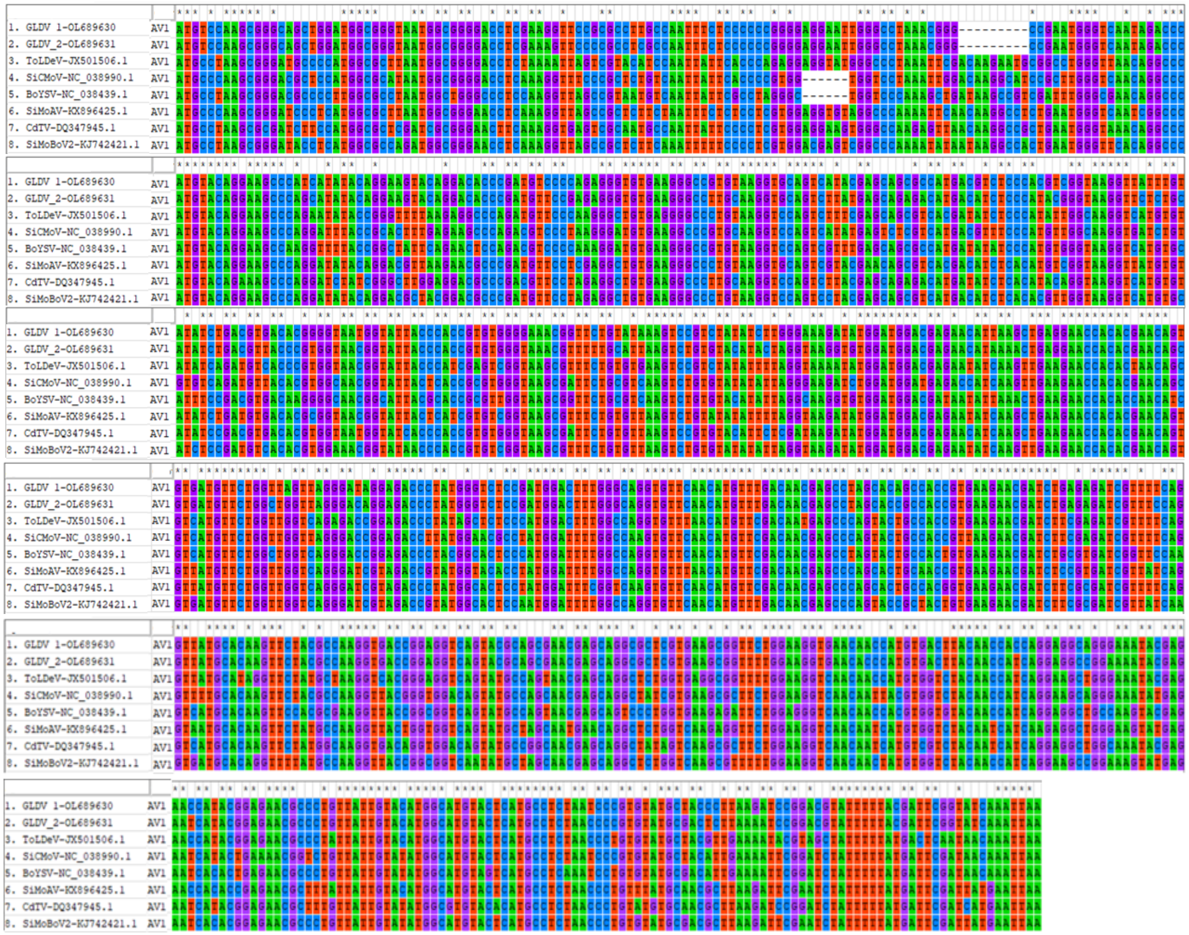

Supplementary Figure 3…continue

AC1 (nucleotide)

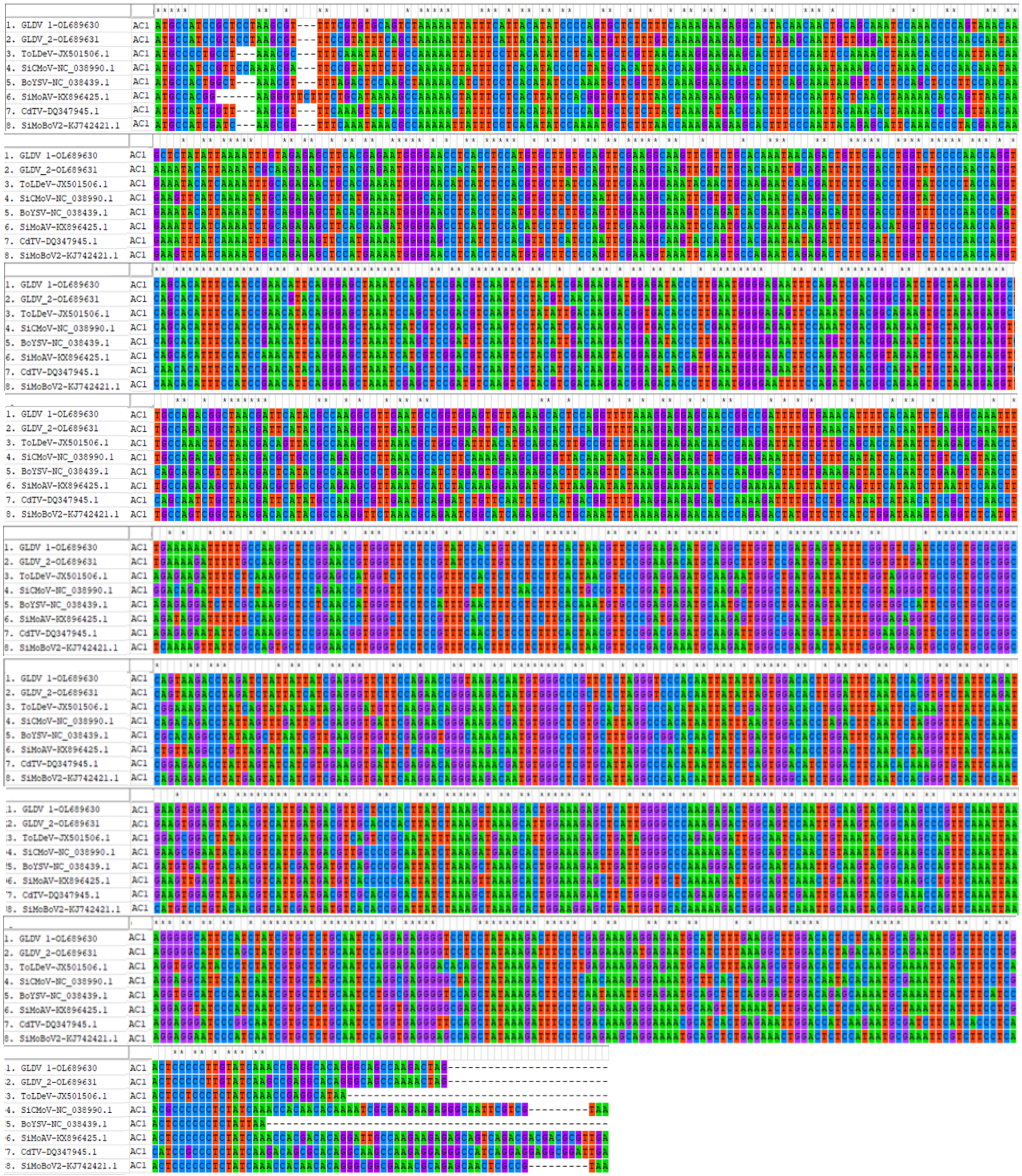

Supplementary Figure 3…continue

AC2 (nucleotide)

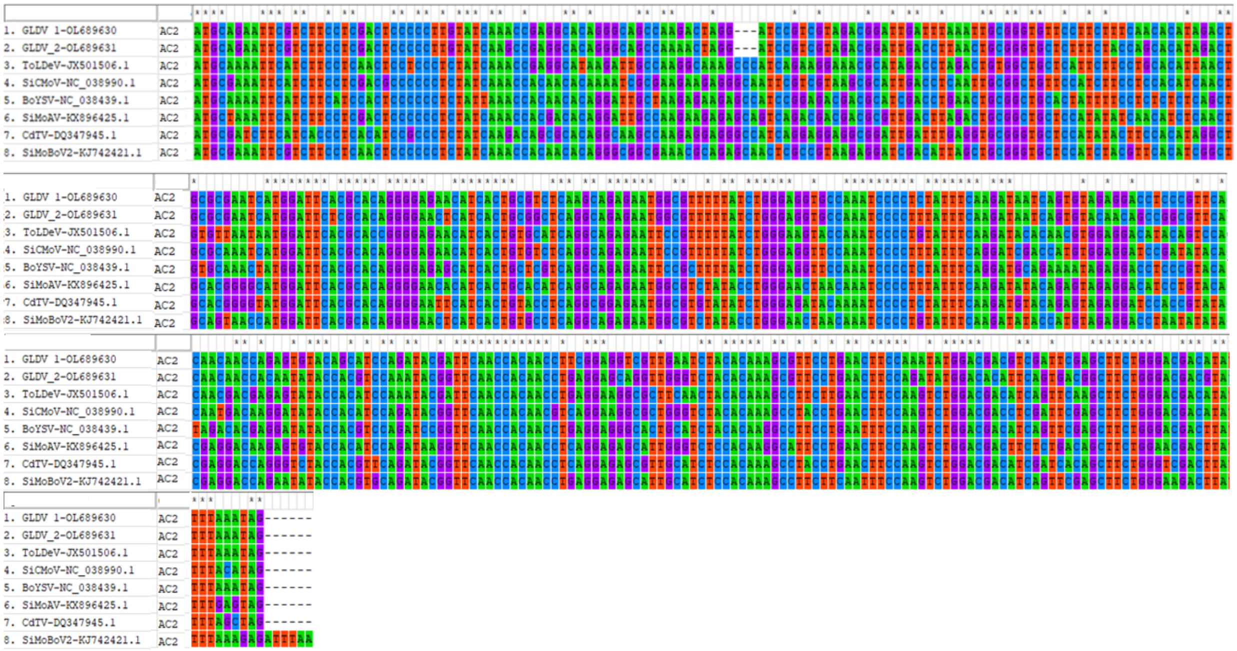

AC3 (nucleotide)

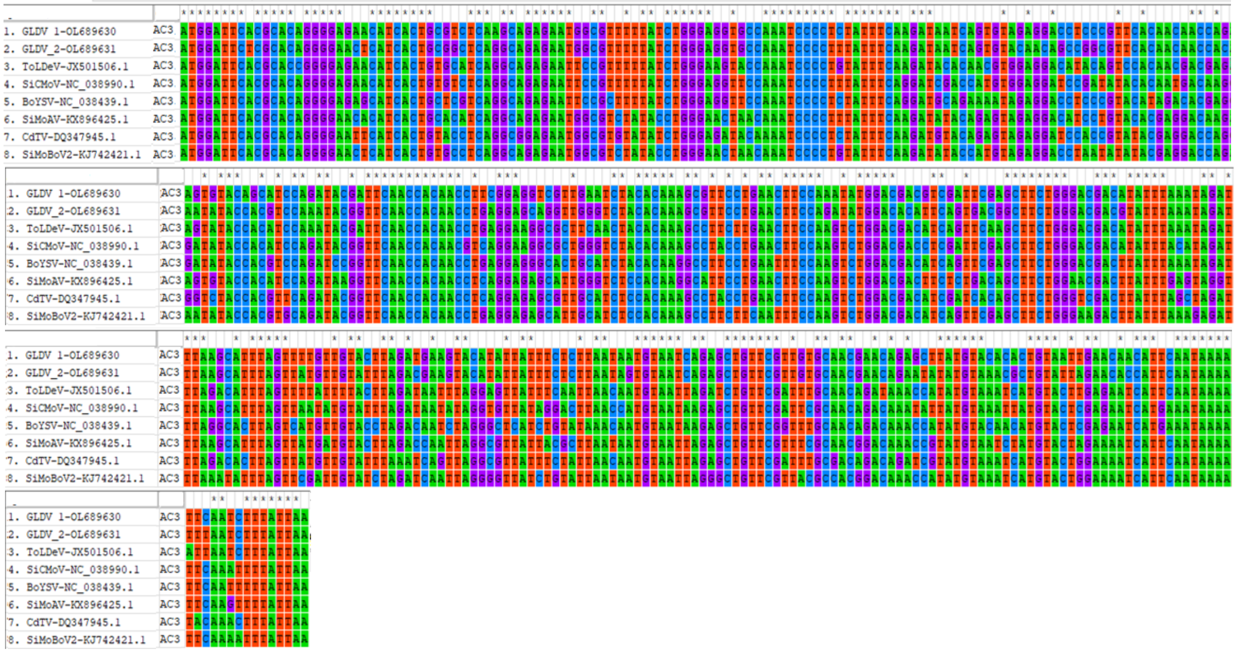

Supplementary Figure 3…continue

AC4 (nucleotide)

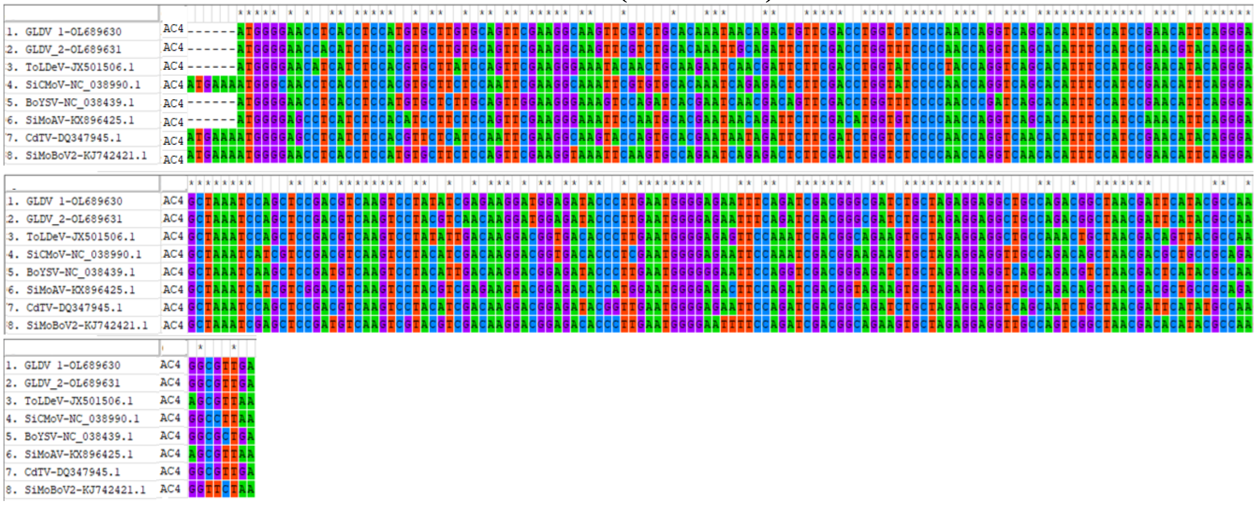

AC5 (nucleotide)

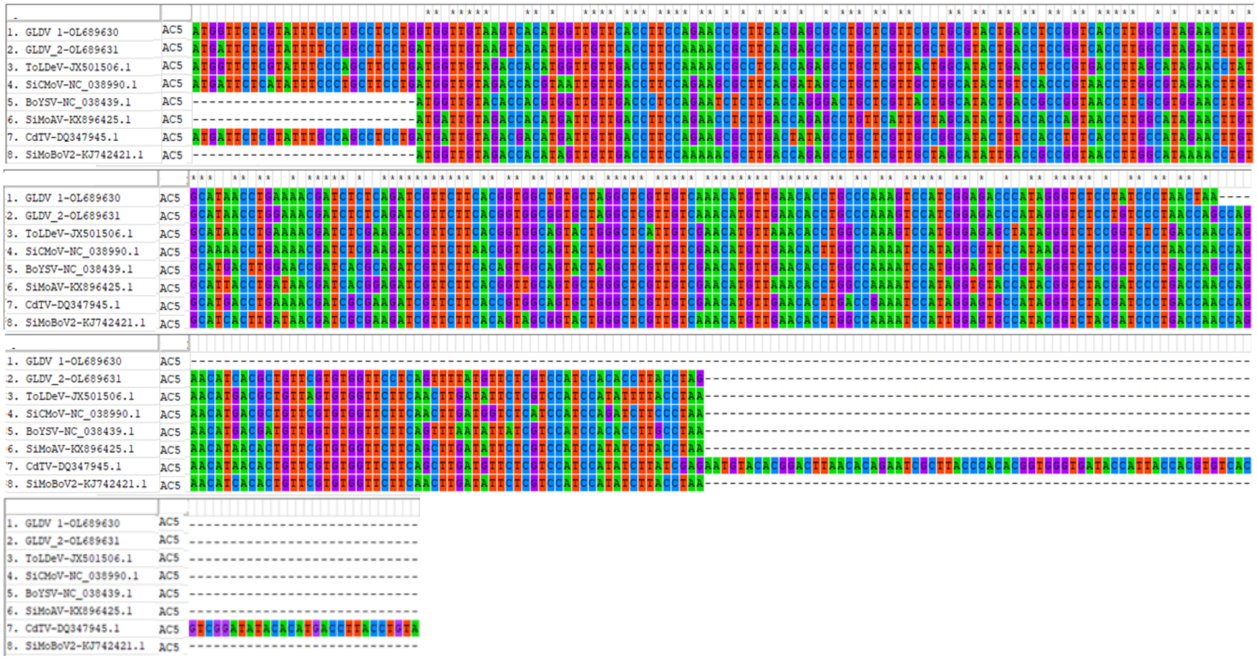

Supplementary Figure 3. Nucleotide alignment of non-coding and coding regions of *Galium leaf distortion virus* (GLDV) isolates and closed related begomoviruses. Alignment of non-coding (Intergenic Region), and coding regions (AV1, AC1, AC2, AC3, AC4, and AC5) nucleotide sequences of GLDV isolates with the corresponding regions of the top six most closely related NW begomovirus DNA-A components was performed with MEGA 7 software and MUSCLE method. Begomovirus acronyms are summarized in **Supplementary Table 1**.

Supplementary Figure 4

AV1 (amino acid)

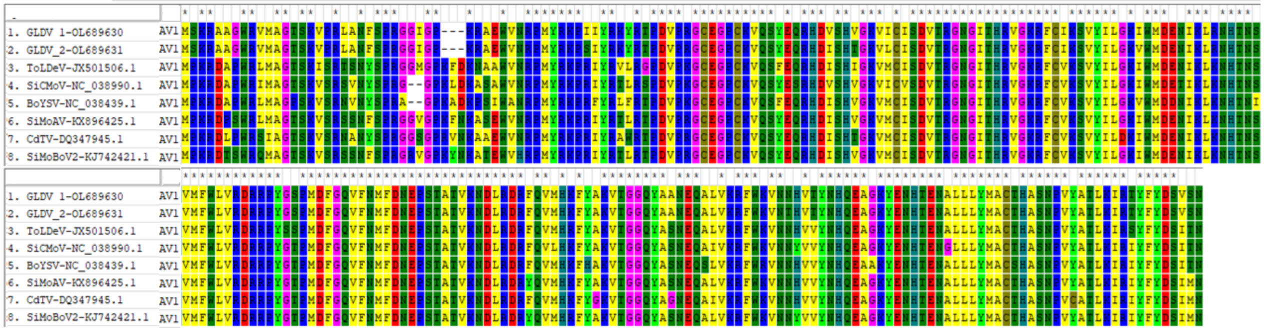

AC1 (amino acid)

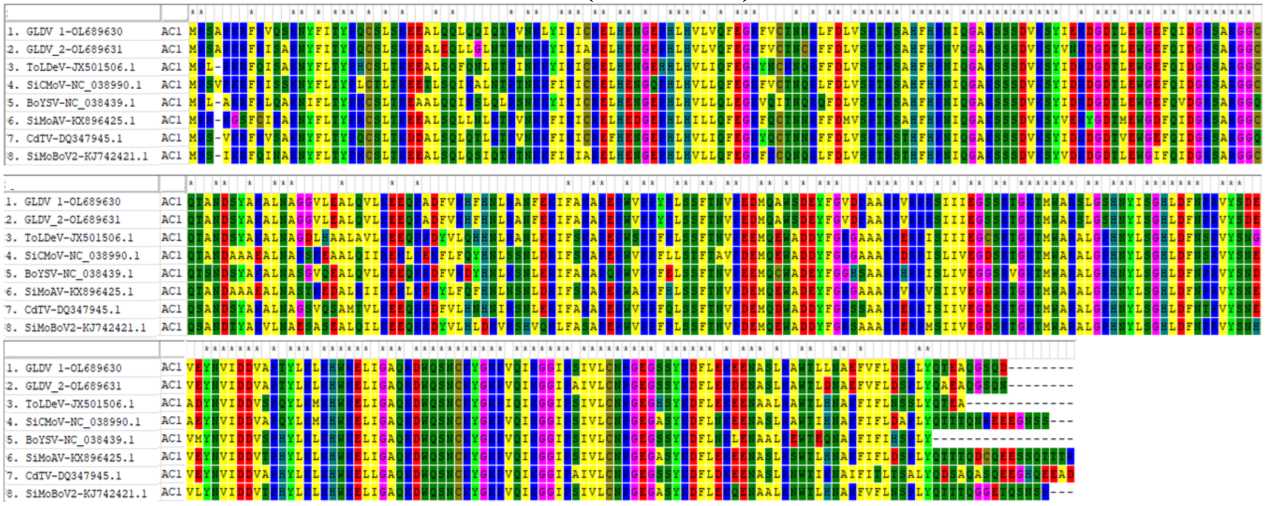

AC2 (amino acid)

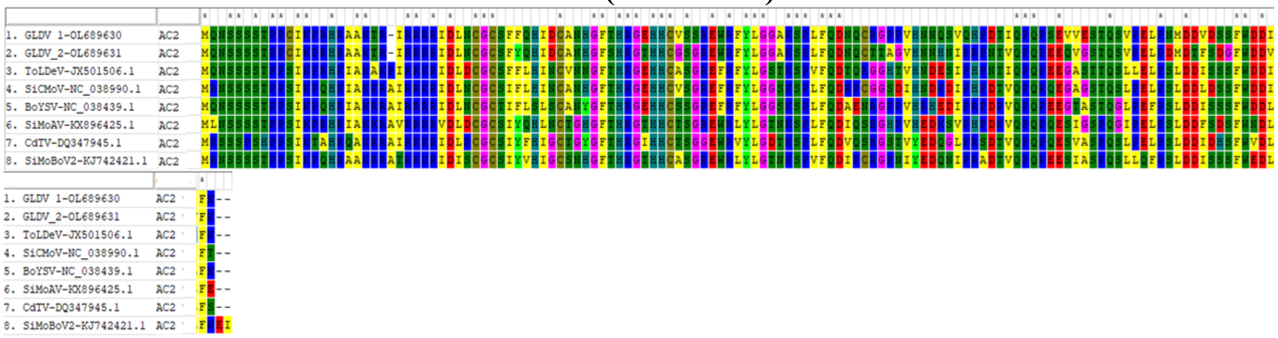

Supplementary Figure 4…continue

AC3 (amino acid)

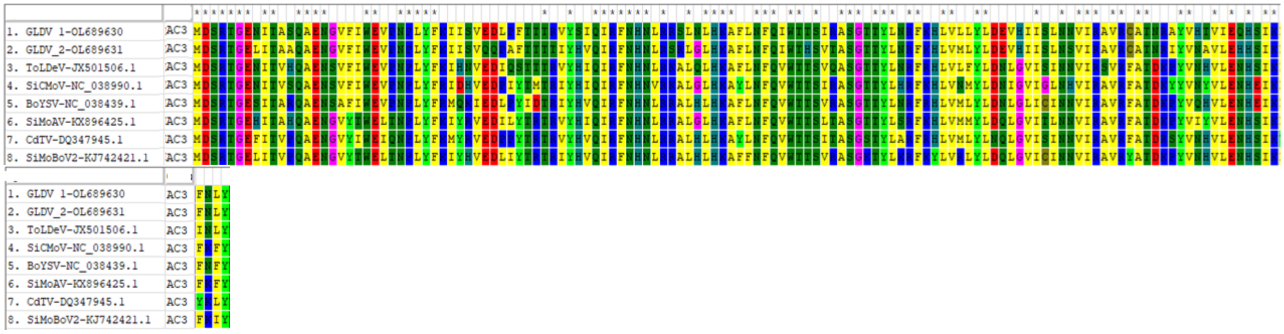

AC4 (amino acid)

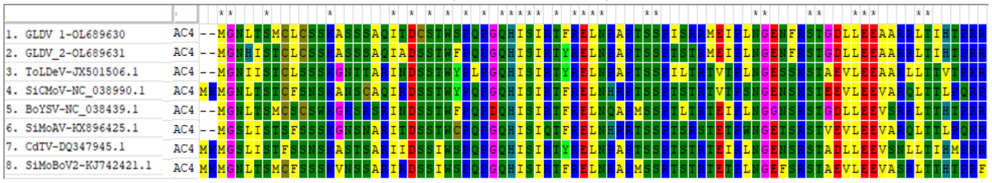

AC5 (amino acid)

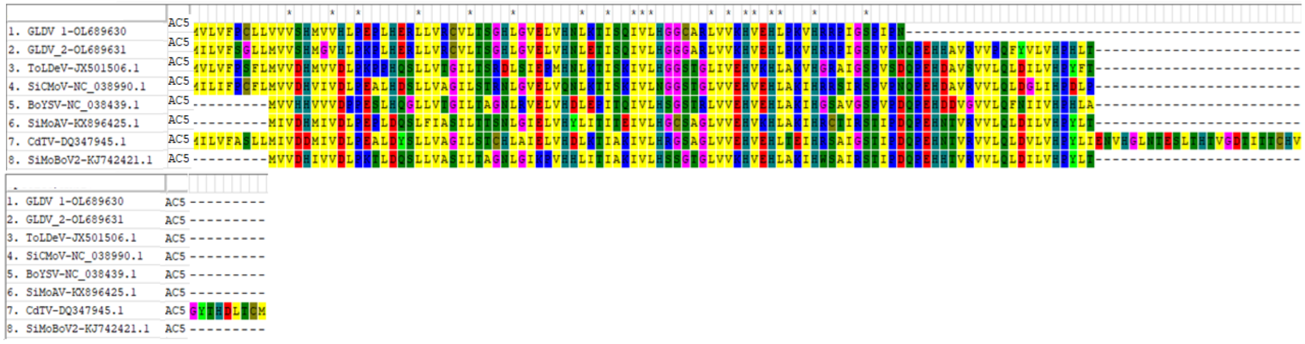

Supplementary Figure 4. Amino acid alignment of coding regions of *Galium leaf distortion virus* (GLDV) isolates and closed related begomoviruses. Alignment of coding regions (AV1, AC1, AC2, AC3, AC4, and AC5) amino acid sequences of GLDV isolates with the corresponding regions of the top six most closely related NW begomovirus DNA-A components was performed with MEGA 7 software and MUSCLE method. Begomovirus acronyms are summarized in **Supplementary Table 1**.

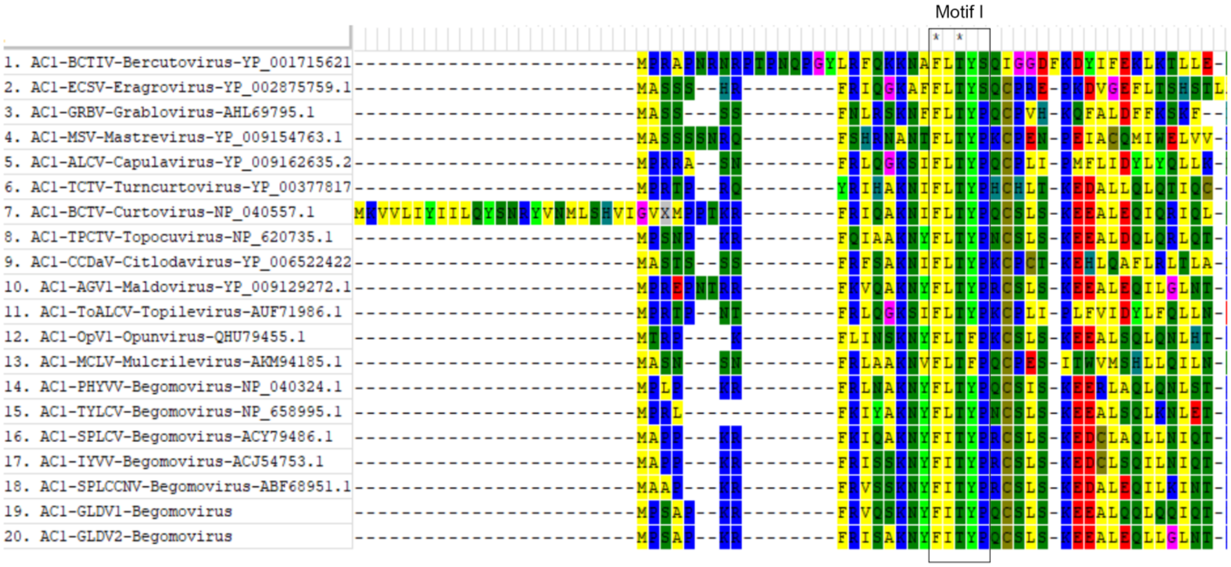

Supplementary Figure 5. *Geminiviridae* family AC1/Rep protein Motif I comparison. N terminus of AC1 protein alignment of representative members of the fourteen genera of *Geminiviridae* family. Amino acid sequence alignment was performed with MEGA 7 and MUSCLE method. Motif I is indicated. Accession numbers of viral isolates are shown. Begomovirus acronyms are summarized in **Supplementary Table 1.**

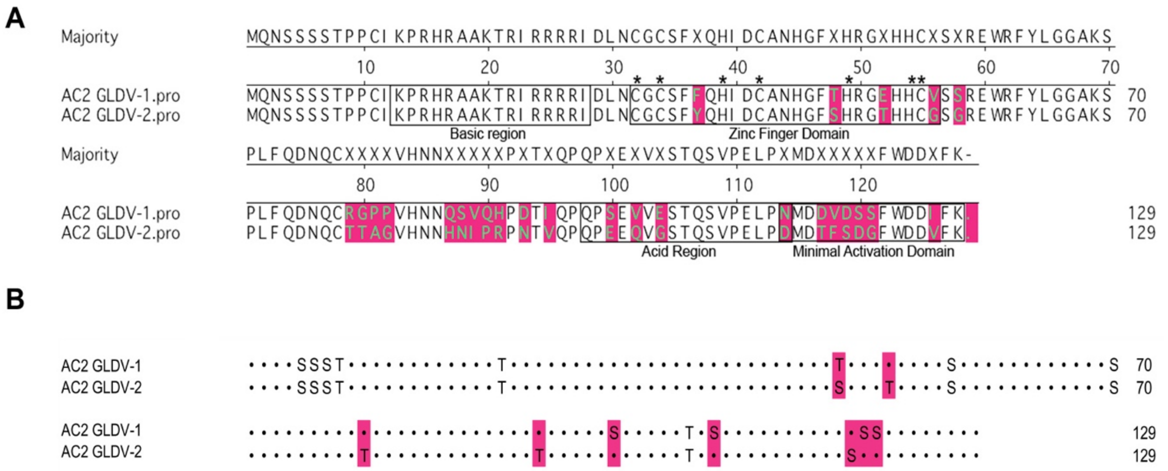

Supplementary Figure 6. *Galium leaf distortion virus (*GLDV) isolates AC2 proteins analysis. **A)** Alignment of GLDV isolates AC2 proteins. AC2 conserved structural features: Basic region, Zinc Finger Domain, Acidic Region, and Minimal Activation Domain, are shown. Conserved cysteine and histidine residues are indicated with black asterisks. **B)** Comparative analysis of putative phosphorylation sites of GLDV isolates AC2 proteins predicted with NetPhos 3.1 Server (https://services.healthtech.dtu.dk/service.php?NetPhos-3.1). Predicted phosphorylated residues Serine (S) and Threonine (T) are shown in sequence alignment. Differential predicted phosphorylated residues are highlighted in magenta. Amino acid residues not predicted as phosphorylation sites are shown as black dots.

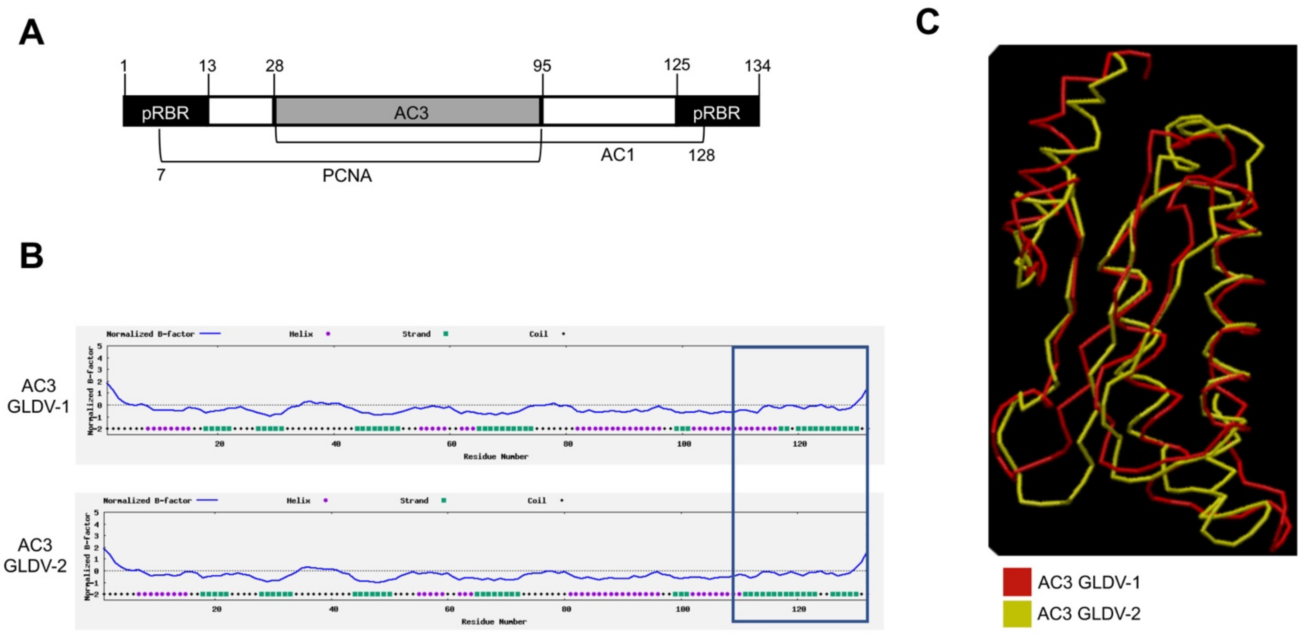

Supplementary Figure 7. ***Galium leaf distortion virus*** **(GLDV) isolates AC3 proteins analysis. A)** AC3 protein interaction regions previously described (Settlage et al., 2005). **B**) The amino acid sequences of GLDV isolates AC3 sequences were ingress into the I-TASSER server (https://zhanggroup.org/I-TASSER/) to determine the content of their secondary structures were attain for each amino acid and build 3D structures. **C**) The 3D structures were compared through SuperPose Version 1.0 tool, (http://superpose.wishartlab.com/). The image was visualized with the option MolScript Superposition Image.
